# Running‐based rehabilitation does not improve muscle function after volumetric muscle loss despite improving hindlimb weight‐bearing asymmetry in mice

**DOI:** 10.1113/EP094201

**Published:** 2026-09-29

**Authors:** Angela S. Bruzina, Molly R. Weberg, Ryan J. Willoughby, Gordon L. Warren, Jarrod A. Call, Sarah M. Greising

**Affiliations:** ^1^ School of Kinesiology University of Minnesota Minneapolis Minnesota USA; ^2^ Department of Physiology and Pharmacology University of Georgia Athens Georgia USA; ^3^ Department of Physical Therapy Georgia State University Atlanta Georgia USA; ^4^ Regenerative Bioscience Center University of Georgia Athens Georgia USA

**Keywords:** loading, neuromusculoskeletal injury, skeletal muscle injury, tibia

## Abstract

Volumetric muscle loss (VML) is a traumatic skeletal muscle injury characterized by chronic functional impairments in the affected muscle and adjacent uninjured bone. Rehabilitative strategies aimed at restoring limb function following VML, including voluntary running, have shown limited efficacy and in some cases have been detrimental. One potential explanation is disproportional loading of the uninjured contralateral limb during running, but evaluation of unilateral weight‐bearing following VML has not been explored. This study was designed to evaluate if the limited response to rehabilitation following VML could be explained by limb loading asymmetry. Twelve‐week‐old C57Bl/6J mice (*n* = 24; equal males/females) were randomized to three groups: Naïve–Run, VML, and VML–Run. Two weeks post‐VML, mice in running groups were provided free access to wheels for 6 weeks. Weight‐bearing asymmetry was assessed before injury and biweekly thereafter. Terminally at ∼20 weeks of age, in vivo muscle function was evaluated, and muscles and bones were saved for analyses. Prior to injury, no difference in weight‐bearing asymmetry was observed across groups. VML‐injured mice exhibited similar weight‐bearing asymmetries prior to running. Following 6 weeks of running, VML–Run mice regained weight‐bearing symmetry comparable to Naïve–Run. Despite this, remaining muscle function remained persistently impaired following VML injury. Collectively this works suggests that voluntary running promotes favourable weight‐bearing adaptations following VML, but does not improve muscle function or the mechanical properties of the adjacent bone.

## Abstract (highlights)

- **What is the central question of the study?**
  Does weight bearing (i.e., loading) change following volumetric muscle loss injuries, and can it explain lack of response in the muscle after rehabilitation?
- **What is the main finding and its importance?**
  Despite improved weight‐bearing asymmetry following volumetric muscle loss, 6 weeks of voluntary wheel running did not improve muscle function. This demonstrates that a lack of loading on the injured limb is not the reason that exercise rehabilitation protocols have been ineffective in restoring function. Further mechanistic evaluation is needed.

## 1 INTRODUCTION

Volumetric muscle loss (VML) is the traumatic loss of skeletal muscle tissue that exceeds the intrinsic regenerative capacity of muscle, resulting in an irrecoverable defect (Grogan et al., 2011). Functional recovery of the remaining musculature is profoundly impaired, and clinically, individuals affected by VML often experience persistent disability (Corona et al., 2015; Garg et al., 2015). Functional deficits are driven, in part, by maladaptive remodelling of the remaining muscle tissue, including excessive fibrotic (Corona et al., 2020; Dolan et al., 2022; Garg et al., 2015; Greising et al., 2017; Hoffman et al., 2022) and lipid deposition (Bruzina et al., 2025, 2026), exacerbated inflammation (Aguilar et al., 2018; Crum et al., 2022; Hurtgen et al., 2016; Larouche et al., 2023) and impaired mitochondrial bioenergetics (McFalin et al., 2023; Southern et al., 2019). Despite apparent functional deficits associated with VML, there are currently no established clinical standards of care for rehabilitation following these injuries.

Preclinical studies have investigated various rehabilitative strategies aimed at recapitulating function of the remaining muscle following VML with limited success. Within the preclinical field, physical rehabilitation strategies, including voluntary wheel running, electrical stimulation, whole body vibration, passive range of motion and combinations of approaches, have been broadly evaluated (Aurora et al., 2014; Basten et al., 2023; Habing et al., 2024; Hoffman et al., 2025; Hu et al., 2022; Johnson et al., 2024; McFalin et al., 2025; Nicholson et al., 2025; Schifino, Raymond‐Pope et al., 2023; Southern et al., 2019; Ziemkiewicz et al., 2023); yet most have been largely ineffective and occasionally, detrimental to functional restoration (Greising et al., 2019). Modest improvements in muscle function with voluntary wheel running following VML have been reported in a few studies (Aurora et al., 2014; Southern et al., 2019), with some deploying a regenerative rehabilitation approach (i.e., regenerative pharmacology and wheel running) to leverage potential synergistic effects (McFalin et al., 2025; Motherwell et al., 2023; Nicholson et al., 2025). Nevertheless, regenerative rehabilitation strategies have yielded a lack of, or minimal functional recovery following VML. The overall mechanisms limiting rehabilitation efficacy following VML have not been clearly elucidated. Responsiveness to rehabilitative intervention following VML does not appear to depend on stimulus intensity, as higher‐load modalities such as resistance (i.e., weighted) wheel running do not improve functional recovery relative to lower‐resistance conditions (Schifino, Raymond‐Pope et al., 2023). Cumulative wheel running distances with VML are comparable to uninjured controls (Aurora et al., 2014; Nicholson et al., 2025; Southern et al., 2019), indicating that overall duration of running does not fully account for the lack of functional restoration.

One potential explanation, specifically for running modalities, is that there may be a disproportionate load on the uninjured contralateral limb while running, thereby limiting effective stimulation of the VML‐injured muscle, although there is a lack of evidence to suggest skeletal muscle hypertrophy in the contralateral limb following VML (Aurora et al., 2014), which would suggest overloading contralaterally. Further, interlimb weight‐bearing with and without running intervention following VML has not been directly evaluated, leaving this mechanism untested.

Disruption to gait biomechanics has been demonstrated following VML and is directly relevant to the translational effectiveness of rehabilitation strategies. Preclinical models of VML exhibit gait abnormalities that closely resemble clinical pathologies (Germanotta et al., 2025), including excessive hip and knee flexion, foot drop and compensatory locomotor adaptations arising from loss of muscle function (Dienes et al., 2019; Eugenis et al., 2022). In addition to altered joint kinematics, indices of loading such as paw contact time and contact area remain impaired chronically after VML injury (Kang et al., 2025; Li et al., 2014). Despite the clinical importance of rehabilitation for functional recovery in patient populations, few preclinical studies have evaluated the effects of rehabilitative interventions on gait biomechanics or weight‐bearing following VML. A short‐term (∼3 weeks) voluntary wheel running intervention initiated 1 week following injury was shown to improve overall gait following VML (Quarta et al., 2017), though it remains unclear whether a more prolonged rehabilitative intervention, independent of regenerative therapies, can influence weight‐bearing following VML injury. Bilateral weight‐bearing has been recently evaluated post‐VML by expressing injured limb loading as a proportion of total force (Kang et al., 2025), but this approach may obscure absolute deficits and potential compensatory strategies of the uninjured limb. Approaches that focus on unilateral performance and asymmetry enable quantification of interlimb imbalances (Obeidat et al., 2023) and can play a critical role in guiding clinical rehabilitative strategies.

The goal of this study was to quantify weight‐bearing asymmetry changes following VML and evaluate whether the lack of response to rehabilitation could be explained by limb loading asymmetry. We hypothesize that regardless of running, functional deficits will persist in the musculoskeletal system of VML‐injured mice. Further, we also hypothesize that there will be no differences in weight‐bearing asymmetry between the running and sedentary groups after VML injury, such that the injured limbs will demonstrate unloading regardless of rehabilitation.

## 2 METHODS

### 2.1 Ethical approval

All protocols were approved by the Institutional Animal Care and Use Committee at the University of Minnesota (2406‐42148A) in compliance with the Animal Welfare Act, the Implementing Animal Welfare Regulations and in accordance with the principles of the *Guide for the Care and Use of Laboratory Animals*.

### 2.2 Experimental design

Adult C57Bl/6J (*n* = 24) mice were purchased from The Jackson Laboratory (no. 000664, Bar Harbor, ME, USA; RRID:IMSR_JAX:000664). Mice were housed on a 12‐h light–dark cycle with *ab libitum* food intake and were given 1 week to acclimate prior to any experimental interventions. At 12 weeks of age, mice (*n* = 16; *n* = 8 females, *n* = 8 males) were randomized to undergo VML injury to the posterior compartment of the left hindlimb or remain injury‐naïve controls (*n* = 8; *n* = 4 females, *n* = 4 males). Fourteen days following injury, VML‐injured mice were randomly assigned to a cage with access to a voluntary running wheel or a standard housing cage to remain sedentary. Rehabilitation was initiated at 2 weeks post‐VML, corresponding to a period of active extracellular and mitochondrial remodelling during which onset timing is thought to influence long‐term outcomes (Aguilar et al., 2018; Hoffman et al., 2022; Larouche et al., 2023; Miller et al., 2025). All injury naïve mice were housed in cages with access to a voluntary running wheel at the same time point. Mice remained with the voluntary wheel or sedentary for 6 weeks, until terminated at 20 weeks of age. Weight‐bearing asymmetry was assessed prior to VML injury (12 weeks of age), prior to voluntary running exposure (14 weeks of age) and biweekly thereafter, for a total of five assessments. Terminally, in vivo skeletal muscle function of the posterior compartment was assessed. The VML‐injured and contralateral gastrocnemius muscles and tibias were dissected and saved for later analyses. Mice were euthanized with pentobarbital (>100 mg/kg, s.c.).

### 2.3 Volumetric muscle loss injury model

Mice underwent a full thickness VML injury to the posterior compartment of the hindlimb, including the gastrocnemius, soleus and plantaris muscles as previously described (Greising et al., 2018; Southern et al., 2019). The VML injury was unilateral, with the left limb as the experimental limb. Approximately 30 min prior to surgery, buprenorphine SR (1 mg/kg, s.c.) was administered for analgesia. Throughout the procedure, 1%–2% isoflurane inhalation was used for anaesthesia and aseptic surgical conditions were maintained. A posterior–lateral incision was created to isolate the posterior compartment and expose the gastrocnemius muscle. A metal plate was inserted between the tibia and deep aspect of the soleus muscle, and a 4‐mm punch biopsy was used on the middle third of the posterior muscle compartment to surgically create the VML defect. The defect piece was weighed (20.9 ± 2.8 mg), and any bleeding was managed with light pressure. The skin incision was sutured. Mice were monitored until ambulatory, then checked twice daily for 72 h post‐surgery and assessed using a standardized clinical scoring system assessing appearance, behaviour, body weight and surgical site integrity. Each assessment category was scored for severity on a scale of 0–3, with 3 being the most severe; total possible score across all categories ranged from 0 to 21. The post‐operative monitoring score reported is the summative score for all assessment categories across each check 72 h post‐VML. Scores were used to confirm lack of pain prior to randomization into sedentary and wheel running groups. All mice recovered from the surgical procedure as expected with no adverse outcomes.

### 2.4 Weight‐bearing asymmetry

Weight‐bearing asymmetry was assessed in all mice prior to any group assignment (∼12 weeks of age), prior to running or sedentary cage assignment (14 weeks of age) and biweekly thereafter. The methodology for assessing weight‐bearing asymmetry was slightly modified (Obeidat et al., 2023), utilizing a custom voluntarily accessed static incapacitance (VASIC) method. Mice were placed in a custom‐built Plexiglas chamber situated on force plates (Static Weight Bearing Touch: Incapacitance Test; BIOSEB, Vitrolles, France). Mice were trained to perform a string‐pulling task (Blackwell et al., 2018) while voluntarily standing on the VASIC platform (Obeidat et al., 2023). Training for the mice consisted of acclimation and habituation. Acclimation was used to familiarize mice with the testing chamber with no other purposeful stimulation. Acclimation consisted of 25 min in the testing chamber, within 5 days of the first and final weight‐bearing assessments at 12 and 20 weeks of age, respectively. Habituation was to acquaint the mice to the string‐pulling task and reward while in their standard housing cage. Habituation sessions consisted of draping 20 strings, which varied in length (53–75 cm), around the perimeter of the standard wire cage hopper. Ten of the strings were randomly baited with a Honey‐Nut Cheerio™ at the end. Habituation was conducted on three occasions throughout the length of the study. First, group‐housed mice were habituated together at ∼12 weeks of age. The following day, mice were habituated individually, within 3 days of the initial weight‐bearing assessment. Finally, all mice underwent the same habituation procedure before weight‐bearing assessment at 16 weeks of age. Weight‐bearing (i.e., loading) was recorded when a mouse had one hindlimb on each load cell while limbs were extended to pull the 100 cm string. Ten readings of weight distribution between hindlimbs were taken per mouse per assessment period, and the average of the 10 data points per limb was used for analysis. Prior to randomization the coefficient of variation of the 10 data points was 13.5% ± 3.9%, which was similar post‐randomization and across the entire study (11.6% ± 4.9%). Weight‐bearing asymmetry was calculated as the difference between weight bearing in grams of each hindlimb, in which a negative asymmetry would represent preferential loading to the injured limb (i.e., left limb).

### 2.5 Rehabilitation with voluntary wheel running

Mice randomized to running rehabilitation were individually housed, with free access to a cage‐mounted wheel (Columbus Instruments, Columbus, OH, USA) equipped with a digital sensor to monitor revolution counts continuously with data aggregation occurring in 10‐min increments. The revolution counts were used to calculate distance run. Data reported was collated for each day as a total run over 24 h, as well as averaged over a week. The total distance run over the 6 weeks was also calculated. With differences in running distances between males and females noted, the data presented are separated by sex.

### 2.6 In vivo plantarflexion muscle function

Terminally at 20 weeks of age (∼8 weeks post‐VML), skeletal muscle function of the posterior compartment of the left hindlimb was evaluated as previously described (Greising et al., 2018; Southern et al., 2019). Briefly, mice were anaesthetized with isoflurane (1–2%) and the left knee was clamped at 90°. The foot of the injured limb was attached to a dual‐mode muscle lever system (300C‐LR; Aurora Scientific, Aurora, Ontario, Canada). Passive torque was assessed across −20° dorsiflexion to 20° plantarflexion joint angles. Next, the anterior compartment of the hindlimb was denervated by severing the common peroneal nerve. The sciatic nerve was stimulated using platinum‐iridium percutaneous needle electrodes across the nerve to elicit a contraction. Isometric torque was assessed at 5, 60, 125, 150, 200 and 250 Hz with 90 s between each frequency. Peak isometric torque was achieved between 150 and 250 Hz and is expressed as millinewton meters per kg of body weight. Averages rates and times to contraction and relaxation were quantified at twitch and peak isometric torque. Correlations between the final weight‐bearing asymmetry assessment and terminal peak isometric torque were evaluated.

### 2.7 Three‐point bending test of tibial bones

Terminally, the tibias were harvested and stored in phosphate‐buffered saline at −20°C until analysis. Tibial bones were analysed by the 3‐point bending test, as previously described (Hoffman et al., 2025; Novotny et al., 2011, 2014; Schifino, Cooley et al., 2023; Warren et al., 1996, 2007). Briefly, the mid‐point (i.e., mid‐diaphysis) of each tibia was measured and marked. For each bone the marked mid‐diaphysis was placed directly under the applied load to measure bone mechanical properties (Mecmesin MultiTest 1‐D, Mecmesin, Horsham, UK; Mecmesin AFG‐25 load cell – 25 N capacity and 5 mN resolution). For each tibia the following data were collected. First, ultimate load, or the maximum force recorded during the test (newtons). Second, stiffness (newtons per millimetre), or the highest slope along the linear portion of the load‐displacement curve prior to ultimate load; the data segment was required to contain at least 50 data points and that the correlation between load and displacement exceed 0.99. Bone‐to‐muscle functional capacity ratios were evaluated by dividing bone ultimate load and stiffness by peak isometric‐torque of the muscle. Finally, ultimate load and stiffness ratios were calculated as the ratio of the injured limb relative to the contralateral.

### 2.8 Biochemical analysis of skeletal muscle

Terminally, the gastrocnemius muscles were harvested and cut into two sections, proximal and distal. Muscles were snap frozen in liquid nitrogen and saved at −80°C for biochemical analyses. The proximal portion of the gastrocnemius were homogenized in phosphate buffer (1:5 ratio; mg/µl) with a phosphatase and protease inhibitor (Thermo Fisher Scientific, Waltham, MA, USA, cat. no. 7844; 1:100 ratio; mg/µl). Muscles were homogenized using 1 mm stainless steel beads (Next Advance, Troy, NY, USA, cat. no. SSB14B) and a tissue homogenizer for ∼5 min, then placed on a nutating mixer for 30 min. Homogenate was centrifuged for 10 min at 10,500 *g* at 4°C, then the supernatant was saved and stored at −80°C. Protein content was analysed in replicate using the Protein A280 setting on NanoDrop One spectrophotometer (Thermo Fisher Scientific) and averaged.

The proximal portion of the gastrocnemius muscles was prepared for immunoblot analyses by separating 20–35 µg of protein by 4%–15% Criterion TGX Stain‐Free Gels (Bio‐Rad Laboratories, Hercules, CA, USA). Following transfer onto a low fluorescence polyvinylidene difluoride membrane, proteins were immunoblotted and detected with the following primary antibodies: 15‐hydroxyprostaglandin dehydrogenase (15‐PGDH; Santa Cruz Biotechnology Biotechnology, Dallas, TX, USA, cat. no. sc271418; 0.2 µg/ml; RRID_10611055), Atrogin‐1 (PhosphoSolutions; Antibodies Inc., Davis, CA, USA, cat. no. AP2041; 1:1000; RRID_2246979) and Akt (Cell Signaling Technology, Danvers, MA, USA, cat. no. 9272; 1:1000; RRID_329827). Primary antibodies were detected using host‐ and isotype‐specific secondary antibodies: anti‐mouse IgG HRP (Cell Signaling Technology, cat. no. 7076; RRID_330924), or anti‐rabbit IgG HRP (Cell Signaling Technology, cat. no. 7074; RRID_2099233). Immunoblots were blocked and incubated using EveryBlot Blocking Buffer (Bio‐Rad Laboratories, cat. no. 13010020) and imagined using a ChemiDoc MP Imaging System (Bio‐Rad Laboratories; RRID:SCR_019037). Bands of interests were identified by the manufacturer‐suggested molecular weight and normalized to total lane protein. Protein expression in each right (uninjured) limb served as an internal normalization control, and the corresponding left (experimental) limb samples were normalized to this value during analysis to allow direct comparison between injured and contralateral limbs. This aligns with the analysis for limb loading.

### 2.9 Statistical analysis

Data were analysed using JMP software (version 18.0, SAS Institute, Cary, NC, USA; RRID:SCR_008567). Longitudinal body weight and average running distance per week were analysed using a linear mixed‐effects model, including experimental group, biologic sex and time point as fixed effects and their interaction (group × sex × time). One‐way ANOVA with Tukey's HSD *post hoc* was used to evaluate differences in running distance between experimental groups across biological sexes. One‐way ANOVA with Tukey's HSD *post hoc* was used to evaluate differences between injury naïve runners, VML‐injured runners and VML‐injured sedentary groups for mechanical properties of the tibia, skeletal muscle function and biochemical outcomes. Student's *t*‐test was used within group for descriptive characteristics, skeletal muscle function, tibial properties, each weight‐bearing assessment and biochemical outcomes to evaluate potential differences between males and females. Post‐operative monitoring score and weight‐bearing asymmetry were analysed using a linear mixed‐effects model, with fixed effects including experimental group, time point and their interaction (group × time) to assess whether symmetry changed over time, differed between groups and whether group differences depended on time. Mouse was included as a random intercept to account for repeated measures across time within animals. Data are presented as means ± SD, and significance was set at *P* ≤ 0.05.

## 3 RESULTS

This project sought to primarily evaluate the impact of VML and rehabilitation on limb loading. We did not expect any differences in loading due to biological sex. Thus, males and females are primarily combined but identified in this work, with few exceptions, and if differences existed, they are noted in the text. Some experimental endpoints are inherently different between males and females, for example muscle and body weight (*t*‐test between males and females within group *P* ≤ 0.003). However, those differences mitigate with normalization to body mass or ratios between limbs. Across other variables not expected to be impacted by sex, statistical evaluations between males and females were conducted (*t*‐test between males and females within group *P* ≥ 0.053). Comparisons between males and females were not intended to address biological sex as a primary outcomes but rather acknowledge when evident.

### 3.1 Longitudinal body weight and running distance

This study evaluated mice that underwent unilateral VML surgery, or were left injury naïve, and then randomized to a rehabilitation intervention of wheel running or remained sedentary. All mice recovered promptly from VML surgery, without complications. Post‐operative monitoring scores in the 72 h following VML surgery were not different between experimental groups (group effect *P* = 0.295), with the maximal scores ranging from 1.0 to 2.5. Scores resolved over time, with greater scores initially post‐VML (time effect *P *< 0.0001). There was no difference between males and females in the monitoring scores (*t*‐test *P* ≥ 0.313). As expected, longitudinal body mass was significantly influenced by biological sex (Figure 1a; sex effect *P *< 0.0001) with males having ∼23% greater body masses across the study. There was no impact of injury or running on body mass (group effect *P* = 0.383). Body mass increased across the 8‐ weeks longitudinally (time effect *P *< 0.0001).

**FIGURE 1:**
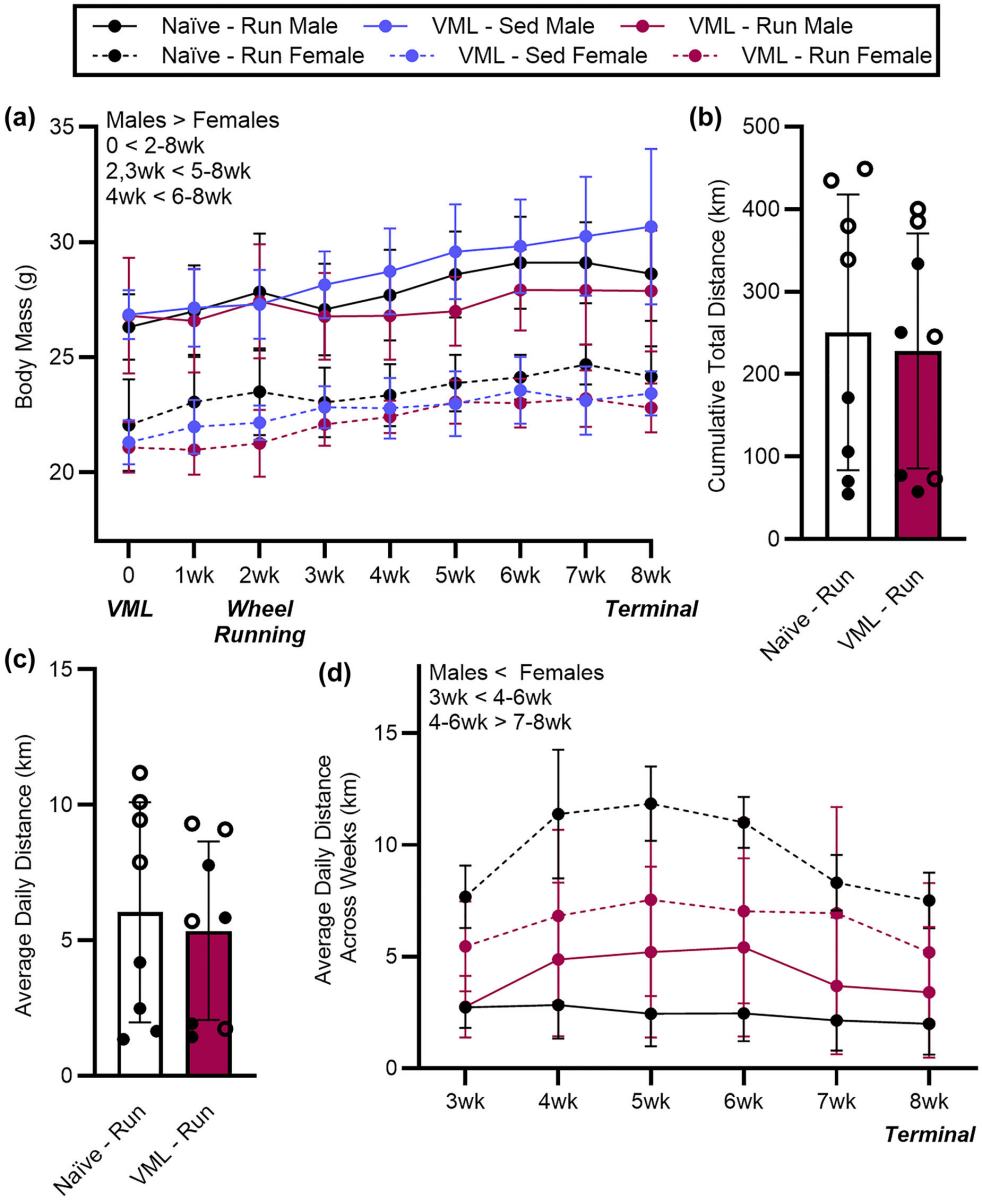
(a) Body weight across 8 weeks (group effect *P* = 0.383; sex effect *P *< 0.0001; time effect *P *< 0.0001; interaction *P* = 0.001), with main effects noted in graph. (b) Cumulative total distance across 6 weeks is not influenced by injury (*P* = 0.776). (c) Injury does not affect the average daily distance covered (*P* = 0.716). Filled circles represent males and open circles represent females; each mouse is shown individually as a data point. (d) Each week, Naïve females covered greater distance daily than Naïve males (group effect *P* = 0.604; sex effect *P* = 0.003; time effect *P *< 0.0001; interaction *P* = 0.005). Data are presented as means ± SD. Bar graphs represent each mouse individually as a data point; line graphs represent average of all mice within a group. Longitudinal body weight and average daily distance by week analysed by linear‐effects mixed model with fixed effects of group, sex and time. Cumulative and average daily distance analysed by one‐way ANOVA; main effects noted on individual graphs.

At 2 weeks post‐injury, mice that underwent VML injury were randomized to cages with free access to a running wheel. All injury‐naïve mice were given access to a running wheel at the same time point. Cumulatively over 6 weeks, total running distance was not significantly influenced by VML injury (Figure 1b; one‐way ANOVA *P* = 0.776). Within a day, the average distance run was ∼6 km, and there was no difference between Naïve and VML groups (Figure 1c; one‐way ANOVA *P* = 0.717). There are reported differences in running distances between males and females (Janowski et al., 2025). In this study, biological sex influenced cumulative running distance in injury‐naïve mice where females covered ∼5 times greater distance than males (*t*‐test *P* = 0.005); however, there was no difference between sexes in VML‐injured mice (*t*‐test *P* = 0.380). For average daily distance, the same pattern emerged with Naïve females covering ∼4 times greater distance than males per day (*t*‐test *P* = 0.002); and there was no difference between sexes in VML‐injured mice (*t*‐test *P* = 0.380). While it has been previously demonstrated that ∼1.5 km/day of wheel running produces localized muscle adaptations (Landisch et al., 2008), it is notable that one male from each experimental group was below this level (∼1.3 and ∼1.4 km/day on average). Average daily distance across weeks was analysed longitudinally (Figure 1d). While VML injury did not influence average daily distance (group effect *P* = 0.604), females, regardless of group, averaged ∼2 times greater running distances across weeks compared to males (sex effect *P* = 0.003). The averaged daily distances across weeks were the greatest in weeks 4, 5 and 6 of experimental time point (time effect *P *< 0.0001).

Masses, ratios, and protein content of hindlimb muscles were evaluated terminally (Table 1). The VML‐injured limb exhibited a ∼24% reduction in gastrocnemius mass (*P* = 0.001), consistent with frank loss of tissue. Antagonist muscles to the gastrocnemius, such as the TA, were not influenced by VML injury or running intervention (*P* = 0.817). Injured to contralateral hindlimb muscle mass ratios were altered with VML injury in the gastrocnemius (*P *< 0.0001), whereas no difference was observed in the TA muscles (*P* = 0.459).

**TABLE 1:** Animal weights and muscle masses.

	Naïve–Run (*n* = 8)	VML–Sedentary (*n* = 8)	VML–Run (*n* = 8)	One‐way ANOVA *P*‐value
Terminal body mass (g)	26.4 ± 2.9	27.1 ± 4.5	25.3 ± 3.3	0.641
Injured (left) TA mass (mg)	51.1 ± 4.1	49.3 ± 10.0	48.6 ± 9.1	0.817
Uninjured (right, contralateral) TA mass (mg)	49.0 ± 4.6	45.6 ± 6.1	49.4 ± 10.5	0.553
Injured:uninjured contralateral TA mass ratio	1.05 ± 0.08	1.07 ± 0.14	1.00 ± 0.13	0.459
Injured (left) gastrocnemius mass (mg)	146.3 ± 15.2	111.1 ± 22.1*	111.6 ± 16.8*	0.001
Injured (left) gastrocnemius mass:body mass (mg/g)	5.55 ± 0.33	4.10 ± 0.38*	4.40 ± 0.46*	<0.0001
Uninjured (right, contralateral) gastrocnemius mass (mg)	145.0 ± 17.2	148.9 ± 16.8	139.9 ± 16.8	0.575
Injured:uninjured contralateral gastrocnemius ratio	1.01 ± 0.04	0.74 ± 0.08*	0.80 ± 0.07*	<0.0001
Injured (left) gastrocnemius protein content (mg/ml)	13.03 ± 1.27	12.63 ± 1.96	11.19 ± 1.50	0.078
Uninjured (right, contralateral) gastrocnemius protein content (mg/ml)	13.40 ± 2.15	13.21 ± 0.75	12.93 ± 2.19	0.872

*Note*: Data are means ± SD. *Significantly different from Naïve–Run.

### 3.2 Voluntary wheel running improves symmetrical limb loading following VML injury

To evaluate the effect of wheel running on weight‐bearing asymmetry, as an indicator of limb loading, in vivo testing of weight distribution between limbs was assessed longitudinally (Figure 2). Within experimental groups at each of the testing times, the majority had no observable differences between biological sexes (*t*‐test *P* ≥ 0.081), except at the 8‐week time point in VML‐injured mice with no running intervention (*t*‐test *P* = 0.011), though there is no clear mechanistic reason for this. There were significant interactions between longitudinal weight‐bearing asymmetry evaluations and experimental group (Figure 2; interaction group × time *P *< 0.0001). Prior to experimental group randomization, there was no difference in weight‐bearing asymmetry between any of the groups. Within the injury‐naïve condition, there was no variation in weight‐bearing asymmetry longitudinally. Following VML, there was an immediate significant impact on weight‐bearing asymmetry, with injured mice loading their uninjured, contralateral limb. Further, VML group randomization caused no difference at this 2‐week time point, prior to randomization to sedentary or running groups. Following VML, sedentarism failed to restore symmetrical weight‐bearing longitudinally, although at the 6‐week time point the variability in this group was high. At the terminal time point the VML sedentary group's weight‐bearing asymmetry was statistically different from both other experimental groups, which were similar. After 6 weeks of running post‐VML, weight‐bearing asymmetry was restored to pre‐injury and injury‐naïve comparable levels.

**FIGURE 2:**
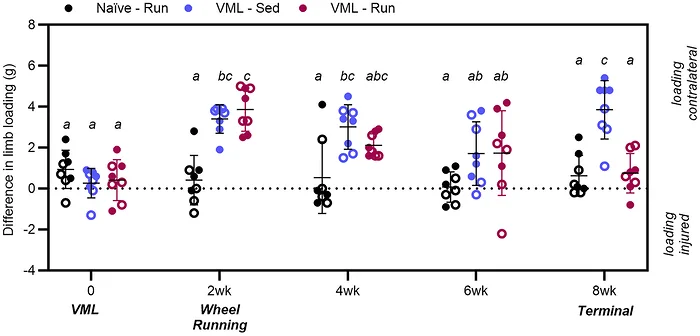
Longitudinal weight‐bearing asymmetry assessments were conducted bi‐weekly in all groups (interaction *P *< 0.0001). Each data point is the average difference between the left and right limbs, and the average of 10 collected loading points during the assessment. With this, 0 g would indicate perfect symmetry between the limbs. Data are presented as means ± SD. Filled circles represent males and open circles represent females; each mouse is shown individually as a data point. Asymmetrical limb loading analysed by linear‐effects mixed model with fixed effects of group and time. Levels that do not share a letter are significantly different.

### 3.3 Voluntary wheel running fails to affect functional capacity of injured limb following VML

To evaluate the effect of wheel running on skeletal muscle function, in vivo functional assessments were performed terminally. Regardless of wheel running intervention, VML injury resulted in ∼47% deficit in maximal torque when normalized to body weight (Figure 3a; *P *< 0.0001) compared to injury naïve. A similar trend in torque production was observed even when normalized to gastrocnemius muscle mass (Figure 3b; *P* = 0.001). Maximal torque was plotted against limb load difference at the terminal time point to determine the relationship between limb asymmetry and function of the remaining muscle. A weak inverse association between limb load difference and isometric torque was observed (Figure 3c; *R* ^2^ = 0.123, *P* = 0.101), suggesting that greater limb asymmetry may be associated with lower maximal force production; however, this did not reach statistical significance. Passive torque was not affected by VML injury or wheel running across plantar‐ and dorsi‐flexion joint angles (Figure 3d; *P* ≤ 0.183). Contractile properties of the injured limb were also evaluated (Table 2). Injury resulted in various impacts to contractile properties of the remaining muscle, independent of running intervention. Average rates of contraction (+d*P/*d*t*) and relaxation (−d*P/*d*t*) were greatly impacted following VML, with ∼50% slower contraction (*P* = 0.007) and relaxation (*P* = 0.001) rates compared to injury naïve. Average rate of contraction was greater in males following VML with running intervention (*t*‐test *P* = 0.003), though there is no clear mechanistic explanation for this. Together, these findings indicate that VML injury substantially impairs both force‐generating capacity and contractile kinetics of the remaining muscle, which are not improved with running intervention.

**FIGURE 3:**
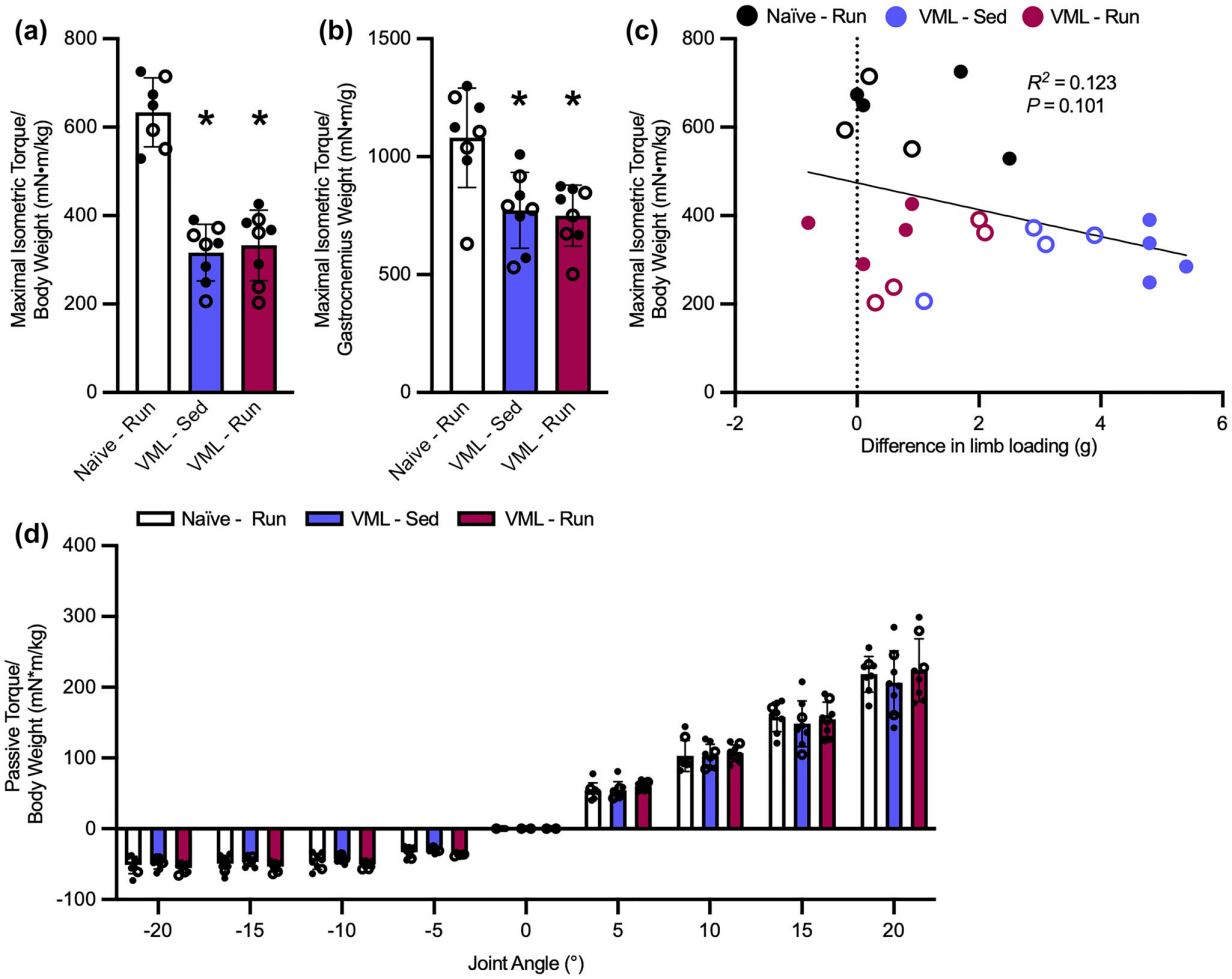
(a) Six weeks of wheel running failed to improve maximal force production normalized to body weight following VML (one‐way ANOVA *P *< 0.0001). (b) Maximal force production normalized to gastrocnemius mass following VML (one‐way ANOVA *P* = 0.001). (c) Relationship between maximal torque and weight‐bearing asymmetry where each point represents an individual mouse; continuous line indicates simple linear regression (*P* = 0.101; *R* ^2^ = 0.123). (d) No influence of VML injury or wheel running on passive torque production across a range of plantar‐ and dorsi‐flexion joint angles of the ankle (one‐way ANOVA at each joint angle; all angles *P* ≥ 0.183). Maximal and passive torque data are presented as means ± SD. Filled circles represent males and open circles represent females; each mouse is shown individually as a data point. *Statistically significantly different from Naïve–Run.

**TABLE 2:** Contractile properties of gastrocnemius muscle.

	Naïve–Run (*n* = 7)	VML–Sedentary (*n* = 8)	VML–Run (*n* = 8)	One‐way ANOVA *P*‐value
Twitch:tetanus ratio	0.176 ± 0.039	0.190 ± 0.035	0.184 ± 0.040	0.771
+d*P*/d*t* (mN m/s)	264.9 ± 99.5	126.2 ± 41.0 *	138.5 ± 54.9 *	0.007
−d*P*/d*t* (mN m/s)	375.3 ± 38.7	194.9 ± 44.5 *	170.7 ± 59.1 *	0.001
Time to peak – twitch (ms)	0.027 ± 0.001	0.024 ± 0.002	0.026 ± 0.003	0.124
Time to peak – maximum (ms)	0.196 ± 0.055	0.200 ± 0.040	0.227 ± 0.036 *	0.023
1/2 RT (ms)	0.094 ± 0.054	0.089 ± 0.041	0.068 ± 0.029 *	0.035

*Note*: Data are means ± SD. *Significantly different from Naïve–Run.

### 3.4 Impact of voluntary wheel running on tibial bone mechanical properties and muscle‐bone functional capacity ratio

Along with the muscle function evaluation terminally, the tibiae from each limb were evaluated for mechanical properties. Average tibia length was 17.4 ± 0.5 mm, and not different between injured and contralateral limbs or across experimental groups (*P* ≥ 0.263). Absolute ultimate load of the injured limb tibia was not affected by VML injury (*P* = 0.281), with an average ultimate load of 12.8 ± 1.5 N across experimental groups. Ultimate load and stiffness of the tibiae were quantified as ratios of the injured limb relative to the contralateral limb. There was no effect of VML injury or wheel running on the ultimate load ratio (Figure 4a; *P* = 0.334) or stiffness ratio (Figure 4b; *P* = 0.994). Tibial ultimate load and stiffness in the VML‐injured limb (i.e., left) were normalized to maximal torque production to account for functional loading of the bone. Ultimate load to torque ratio was ∼91% greater with VML injury, regardless of wheel running intervention (Figure 4c; *P* = 0.002). Similarly, stiffness to torque ratio was influenced by VML injury alone, exhibiting ∼2 times greater stiffness compared to injury naïve (Figure 4d; *P* = 0.001). There was no relationship between ultimate load ratio and weight‐bearing asymmetry (Figure 4e; *R* ^2^ = 0.0009, *P* = 0.885).

**FIGURE 4:**
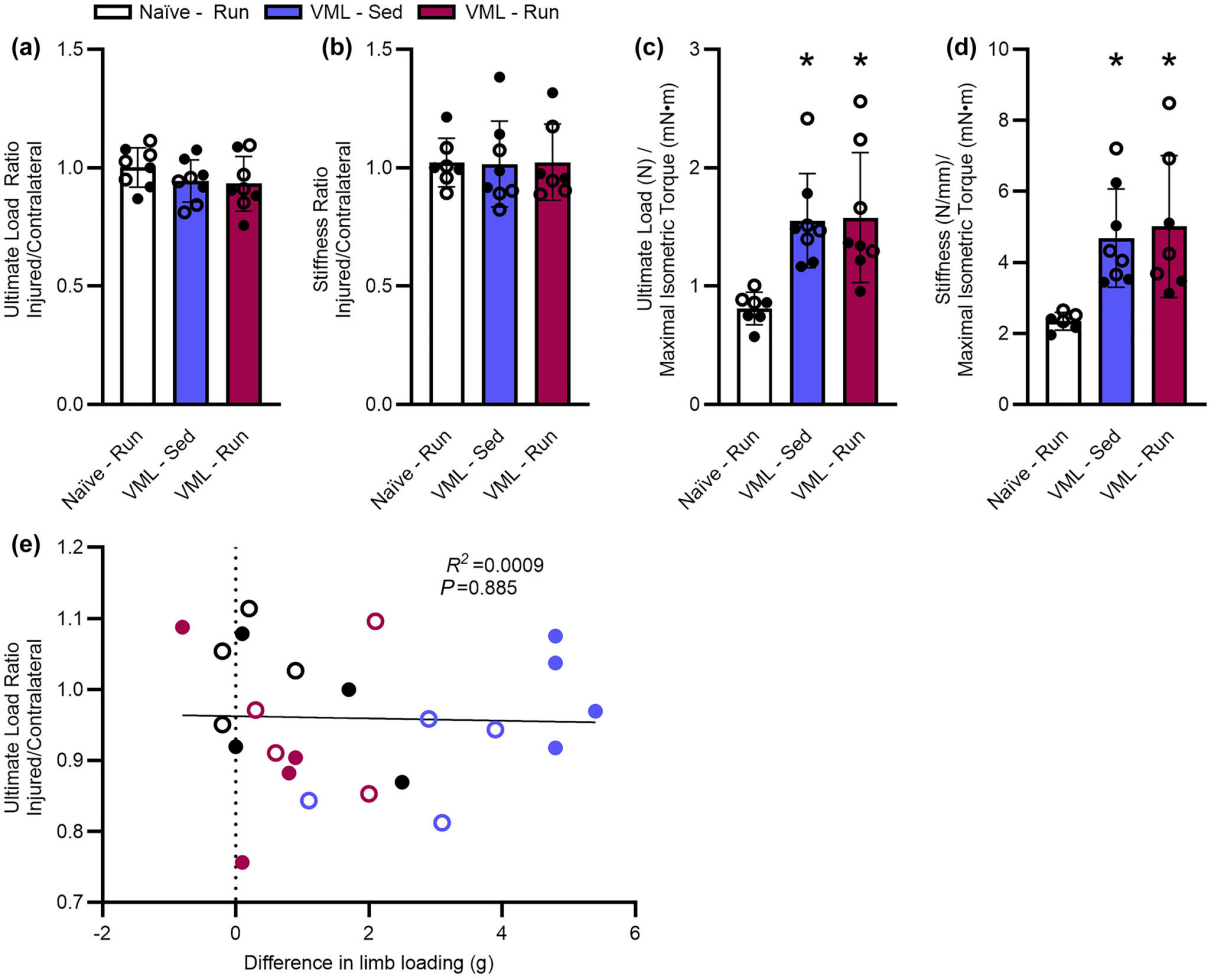
(a) Ratio of ultimate load between injured and contralateral limb (*P* = 0.334). (b) Ratio of stiffness between injured and contralateral limb (*P* = 0.994). (c) Ultimate load normalized to absolute maximal isometric torque (*P* = 0.002). (d) Stiffness normalized to absolute maximal isometric (*P* = 0.001). (e) Relationship between ultimate load and weight‐bearing asymmetry where each point represents an individual mouse; continuous line indicates simple linear regression (*P* = 0.885; *R* ^2^ = 0.0009). Ultimate load and stiffness data are presented as means ± SD. Data analysed by one‐way ANOVA. Filled circles represent males and open circles represent females; each mouse is shown individually as a data point. *Statistically significant different from Naïve–Run.

### 3.5 Signalling across hindlimbs in the remaining muscle is not influenced by voluntary wheel running

To determine whether inflammatory, atrophic or hypertrophic signalling may explain the restoration of symmetrical limb loading with wheel running following VML, protein expression was evaluated in injured and uninjured contralateral gastrocnemius muscles (Figure 5a). Values are reported as the ratio of injured (left) to uninjured contralateral (right) gastrocnemius muscle protein expression, and there was a similar response in both VML‐injured groups. Expression of 15‐PGDH, an enzyme involved in dampening inflammatory signalling, was ∼3 times greater in the VML‐injured gastrocnemius relative to the contralateral compared with the ratio observed in injury naïve (Figure 5b; *P* = 0.001). The ratio of Atrogin‐1 expression, a marker of skeletal muscle atrophy, was ∼33% lower following VML injury (Figure 5c; *P* = 0.008), indicating reduced expression in the VML‐injured gastrocnemius relative to the contralateral. In contrast, the ratio of Akt expression, a marker of cellular growth and metabolic signalling, was ∼3 times greater with VML injury (Figure 5d; *P* = 0.002), reflecting increased expression in VML‐injured muscle. Signalling in the remaining muscle following VML is impacted independent of voluntary wheel running, where proliferative pathways may be prioritized while catabolic and inflammatory pathways may be suppressed.

**FIGURE 5:**
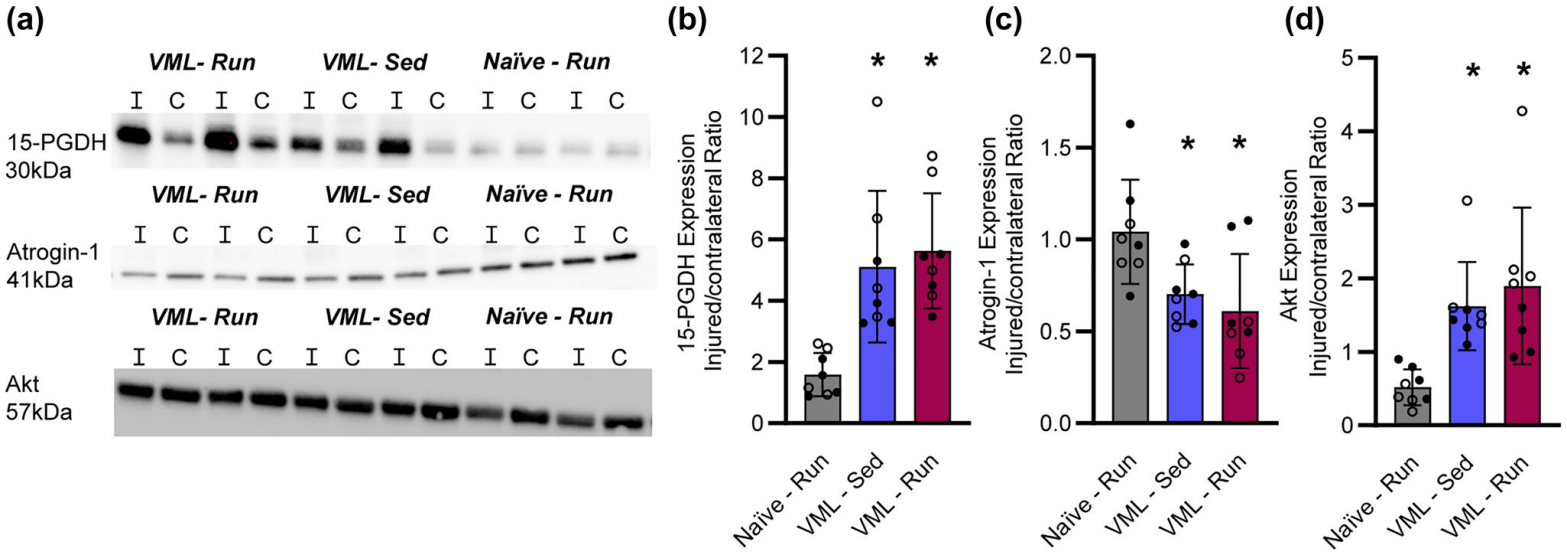
(a) Chemiluminescence blots for quantification of band intensity for 15‐PGDH, Atrogin‐1 and Akt in injured (I) and uninjured contralateral (C) gastrocnemius muscles. (b) Ratio of 15‐PGDH expression between gastrocnemius muscles was greater post‐VML, regardless of wheel running (*P* = 0.001). (c) Ratio of Atrogin‐1 expression between gastrocnemius muscles decreased following VML (*P* = 0.008). (d) Ratio of Akt expression between gastrocnemius muscles was greater following VML (*P* = 0.002). Data are presented as means ± SD. Filled circles represent males and open circles represent females; each mouse is shown individually as a data point. Data analysed by one‐way ANOVA. *Statistically significant difference from Naïve–Run.

## 4 DISCUSSION

This study investigated the impact of a 6‐week running intervention on hindlimb weight asymmetry following VML, as well as neuromuscular function, bone function and muscle protein content. To the best of our knowledge, weight‐bearing symmetry has not been previously measured following VML and rehabilitative exercise interventions. Herein, 6 weeks of voluntary wheel running restored hindlimb weight symmetry following VML but failed to restore active force generation of the remaining muscle. The mechanical properties of the tibia, both adjacent and contralateral to the VML injury, are not effectively influenced by voluntary wheel running. One acknowledged limitation of the current study is the lack of an injury‐naïve sedentary experimental group, and consideration should be given to this when interpreting significant effects from wheel running or VML injury.

Our findings align with broader evidence indicating voluntary wheel running consistently fails to influence maximal torque produced by the remaining muscle following VML. Across preclinical species, most voluntary rehabilitation protocols are initiated at least 1 week post‐VML and are maintained for a minimum of 6 weeks (Aurora et al., 2014; Corona et al., 2013; Motherwell et al., 2023; Nicholson et al., 2025; Quarta et al., 2017; Schifino, Raymond‐Pope et al., 2023). The experimental design herein is initiated in a similar manner, starting 2 weeks following injury and continuing for 6 weeks. Rehabilitation initiated at this time point corresponds to a period of active extracellular matrix remodelling, mitochondrial dysfunction and inflammatory signalling, processes that evidence suggests are important to long‐term outcomes following VML (Aguilar et al., 2018; Hoffman et al., 2022; Larouche et al., 2023; Miller et al., 2025). Clinically, initiation of rehabilitation acutely following soft tissue injury has been shown to shorten the time to pain‐free activity (Bayer et al., 2017). Aggressive rehabilitation for patients following VML, including early weight bearing, has shown promise in improving lower extremity strength (Blair et al., 2016). However, there remain few preclinical studies demonstrating that early rehabilitation approaches are effective or translatable to the clinic. Preclinical models of VML injury appear to resume baseline wheel running activity by 1 week following injury. Despite this, some studies have shown that early wheel running may be detrimental to muscle regeneration following VML, with greater fibrosis and smaller myofibre cross sectional area (Quarta et al., 2017).

Herein, injury‐naïve and VML‐injured running groups exhibited comparable rehabilitation volume, and mice met the expected running threshold per day recognized to induce positive adaptations to skeletal muscle (Landisch et al., 2008). Mixed evidence in the field is suggestive of whether wheel running adequately loads the injured limb and thus results in hypertrophy of injured muscle mass (Aurora et al., 2014; Motherwell et al., 2023). Seven weeks of voluntary wheel running increased mass and hypertrophic (e.g., IGF‐1) signalling in uninjured rat TA muscle, which could suggest that the uninjured limb was being favoured (Motherwell et al., 2023), although comparable fibre cross‐sectional area between uninjured and injured rat TA muscle has been reported (Aurora et al., 2014), which implicates adequate loading of both limbs. Restoration of muscle mass does not consistently translate to improved functional outcomes, and thus anticipated adaptations from rehabilitation alone are insufficient to overcome the underlying deficits that ultimately limit functional recovery after VML. Further, while lack of loading the injured limb is plausible, this would likely induce adverse changes in bone quality and subsequent mechanical properties of the bone, which is not observed herein. Histological evaluation of muscle following VML and voluntary wheel running interventions is still needed to understand regenerative and repair processes. Rehabilitative approaches, such as wheel running, have the potential to enhance the remaining muscle following VML to improve metabolism, vascularization, innervation and activation of satellite cells. Future work should couple histological evidence with evaluations of loading and muscle strength.

The absence of elevated passive torque in VML‐injured animals across the range of joint angles assessed differs from prior reports that documented greater passive stiffness following VML injury (Hoffman et al., 2025; Schifino, Cooley et al., 2023). This discrepancy is most likely methodological: prior studies assessed passive stiffness at a single fixed dorsiflexion angle of 20°, whereas passive torque here was characterized across a plantar‐ to dorsi‐flexion range, which captures a different dimension of passive joint behaviour and is less sensitive to the localized stiffening produced by fibrotic deposition. The contribution of fibrotic remodelling to passive mechanical properties across joint angle ranges warrants direct comparison in future work. VML injury also substantially impaired the kinetics of force generation and relaxation in the remaining muscle as rates of contraction and relaxation were approximately 50% slower compared with Naïve–Run animals, independent of running intervention (Table 2). These contractile kinetics reflect the dynamics of cross‐bridge cycling and calcium handling within the remaining muscle fibres, processes that can be disrupted by the fibrotic infiltration, metabolic dysfunction and denervation that characterize VML pathophysiology (Garg et al., 2015; Greising et al., 2017; Hoffman et al., 2022; Sorensen et al., 2021). That voluntary wheel running did not improve contractile kinetics reinforces the conclusion that the structural and metabolic barriers to functional recovery after VML are not completely resolved by loading‐based rehabilitation.

Although restoration of limb function remains a primary outcome in studies evaluating voluntary wheel running following VML, interpretation of torque measurements is influenced by method of normalization. Here, there was no effect of wheel running on the mass of injured gastrocnemius or the mass ratio in respect to the contralateral, which aligns with the lack of observed differences in muscle function when normalized by either body weight or injured muscle mass. There appears no consensus on which normalization factor is most appropriate and, at minimum, reporting torque normalized to both body weight and injured muscle mass may facilitate a more comprehensive interpretation of functional recovery with rehabilitative intervention following VML. In vivo torque production is commonly normalized to total body weight, thereby enabling more appropriate comparisons of muscle function between subjects (Call et al., 2025). However, prolonged voluntary wheel running frequently results in significantly lower total body weight relative to injury‐matched sedentary groups, potentially influencing normalized functional outcomes independent of adaptations within the injured muscle. Given the substantial loss of muscle mass characteristic of VML injury, it could be asked whether normalizing to the remaining musculature provides a more appropriate assessment of force‐generating capacity. Even within individual studies (Corona et al., 2013; McFalin et al., 2025; Schifino, Raymond‐Pope et al., 2023), investigating the torque normalized to body weight and injured muscle mass following voluntary running have yielded discrepant outcomes. Normalization factor may be particularly relevant with regenerative rehabilitative strategies and has implications for therapeutic strategies. For example, treatment with β‐adrenergic receptor agonist (e.g., formoterol), in combination with wheel running (McFalin et al., 2025), increased gastrocnemius mass relative to total body mass; however, this did not translate into meaningful improvements in function.

Voluntary wheel running has shown promise in favourably impacting the bone adjacent to VML injury; therefore, the mechanical properties of both adjacent and contralateral tibiae were evaluated. Here, neither VML injury nor wheel running altered the stiffness or ultimate load ratios between adjacent and contralateral tibia. These findings contrast with our own previous work demonstrating reduced ultimate load in the adjacent bone to VML compared with uninjured controls (Schifino, Cooley et al., 2023). Several factors may explain the discrepancy, including being underpowered to detect modest differences in bone mechanical properties. Alternatively, it is plausible that repeated exposure to the weight‐bearing testing throughout the study may have provided sufficient osteogenic stimulus to attenuate bone losses in the injured limb. Across the experiment, VML‐injured mice accumulated 80–155 min of habituation and weight‐bearing assessments, during which they were repeatedly encouraged to stand on their hindlimbs. A similar phenomenon has been reported in a hindlimb suspension model, where targeted mechanical loading of the tibia for 4 weeks preserved bone mineral content and mechanical properties of an otherwise substantially unloaded limb (Yang et al., 2018). Repeated hindlimb weight‐bearing may serve as a potential rehabilitation strategy to preserve bone following VML and warrants further investigation. Despite persistent weight‐bearing asymmetry in VML‐injured mice without wheel access, repeated weight bearing alone may be sufficient to protect the adjacent bone. Voluntary wheel running appears to provide little, if any, additional benefit to tibial mechanical properties. The independent contribution of voluntary wheel running in preserving the adjacent bone following VML, in the absence of the weight‐bearing stimulus, remains unknown.

Importantly, although tibial mechanical properties did not differ across groups in absolute or within‐animal terms, the bone‐to‐muscle functional capacity ratio was approximately 91% greater in VML‐injured animals compared with naïve runners, independent of wheel running intervention (Figure 4c). This elevated ratio reflects the well‐characterized musculoskeletal mismatch following VML, wherein muscle function deteriorates disproportionately relative to bone strength. Schifino, Cooley et al. (2023) first documented this mismatch in this model, and Hoffman et al. (2025) subsequently demonstrated that low‐intensity vibration training could partially improve bone outcomes without resolving the underlying muscle deficit. The present data indicate that voluntary wheel running, despite normalizing limb loading asymmetry, does not resolve this bone‐muscle functional imbalance.

The difference between the present bone findings and those of Hoffman et al. (2025) is important to consider mechanistically. Vibration training at 1.0 g improved tibial ultimate load by approximately 16% in VML‐injured mice; voluntary wheel running produced no comparable benefit here. The present data offer a direct mechanistic reason, that is, VML‐injured mice unloaded the injured limb across the study period, as evidenced by persistent weight‐bearing asymmetry in sedentary VML‐injured animals and its resolution only after 6 weeks of running. If the injured limb receives reduced mechanical loading during voluntary locomotion, it is unlikely to accumulate the strain necessary to drive cortical bone adaptation. Vibration training bypasses this constraint by delivering a direct oscillatory mechanical stimulus to bone independent of voluntary loading behaviour, which may account for its osteogenic efficacy where running rehabilitation fails.

Interpretation of these mechanical findings is limited herein by the absence of bone microstructural data. In both prior studies in this model, cross‐sectional moment of inertia and periosteal diameter were the primary structural determinants of tibial ultimate load changes after VML (Hoffman et al., 2025; Schifino, Cooley et al., 2023); without micro‐computed tomography, it cannot be determined whether those structural parameters are altered in the present cohort, nor whether the weight‐bearing assessments preserved bone geometry as well as whole‐bone mechanical function. Finally, the inclusion of both male and female mice, while expanding the translational scope of these findings, introduces an additional interpretive consideration for bone outcomes specifically. Prior work in this model used exclusively male mice, and sex‐based differences in bone geometry and strength may have increased within‐group variability sufficiently to reduce power to detect injury‐related differences in ultimate load. Whether females exhibit the same VML‐induced bone structural and mechanical deficits established in male mice remains an open question with direct clinical relevance.

### 4.1 Conclusion

Despite limited adaptations due to rehabilitation in the muscle or bone, the mitigation of limb loading asymmetry after 6 weeks of voluntary running is promising. These findings reinforce the idea that limb loading and gait do not necessarily reflect the true functional ability of the entire musculoskeletal unit. Normalization of limb loading and gait may instead arise due to compensatory strategies that allow for movement despite the continued functional deficits. When considered alongside the rest of the literature concerning VML and gait and limb loading patterns, this work highlights a critical gap between biomechanical improvement and functional recovery following treatments including rehabilitative interventions. Notably, even regenerative therapies capable of producing greater structural restoration than rehabilitation alone have demonstrated only modest improvements in gait mechanics, with persistent locomotor abnormalities indicating incomplete recovery of limb function (Dziki et al., 2017; Eugenis et al., 2022; Lyu et al., 2026), underscoring that this disconnect between biomechanical and functional improvement is not unique to running‐based rehabilitation. Addressing this disconnect will be useful for advancing VML therapies and promoting interventions that restore muscle function alongside whole limb function.

## AUTHOR CONTRIBUTIONS

Angela S. Bruzina and Molly R. Weberg contributed equally to this work as co‐first authors. Conception or design of the work: Sarah M. Greising and Jarrod A. Call. Acquisition, analysis, or interpretation of data for the work: Angela S. Bruzina, Molly R. Weberg, Ryan J. Willoughby, Gordon L. Warren, Jarrod A. Call, and Sarah M. Greising. Drafting of the work or revising it critically for important intellectual content: Angela S. Bruzina, Molly R. Weberg, Jarrod A. Call, and Sarah M. Greising. All authors approved the final version of the manuscript. All authors agree to be accountable for all aspects of the work in ensuring that questions related to the accuracy or integrity of any part of the work are appropriately investigated and resolved. All persons designated as authors qualify for authorship, and all those who qualify for authorship are listed.

## CONFLICT OF INTEREST

None declared.

## GENERATIVE AI STATEMENT

No AI tools were used in this work.

## Data Availability

The datasets used and/or analysed during the current study are primarily presented in the current manuscript and are available from the corresponding author on request.
